# Evaluation of Physicochemical Properties of Ipsapirone Derivatives Based on Chromatographic and Chemometric Approaches

**DOI:** 10.3390/molecules29081862

**Published:** 2024-04-19

**Authors:** Wiktor Nisterenko, Damian Kułaga, Mateusz Woziński, Yash Raj Singh, Beata Judzińska, Karolina Jagiello, Katarzyna Ewa Greber, Wiesław Sawicki, Krzesimir Ciura

**Affiliations:** 1 Department of Physical Chemistry, Faculty of Pharmacy, Medical University of Gdańsk, Aleja Generała Józefa Hallera 107, 80-416 Gdańsk, Poland; wnisterenko@gmail.com (W.N.); mateusz.wozinski@gumed.edu.pl (M.W.); katarzyna.greber@gumed.edu.pl (K.E.G.); w.sawicki@gumed.edu.pl (W.S.); 2 Department of Organic Chemistry and Technology, Faculty of Chemical Engineering and Technology, Cracow University of Technology, 24 Warszawska Street, 31-155 Cracow, Poland; damian.kulaga@pk.edu.pl; 3 Department of Pharmaceutical Quality Assurance, LJ Institute of Pharmacy, LJ University, Ahmedabad 382210, India; yashraj.0804@gmail.com; 4 QSAR Lab, Trzy Lipy 3, 80-172 Gdańsk, Poland; b.judzinska@qsarlab.com (B.J.); k.jagiello@qsarlab.com (K.J.); 5 Laboratory of Environmental Chemoinformatics, Faculty of Chemistry, University of Gdansk, Wita Stwosza 63, 80-308 Gdansk, Poland

**Keywords:** ipsapirone derivatives, lipophilicity, QSAR, machine learning

## Abstract

Drug discovery is a challenging process, with many compounds failing to progress due to unmet pharmacokinetic criteria. Lipophilicity is an important physicochemical parameter that affects various pharmacokinetic processes, including absorption, metabolism, and excretion. This study evaluated the lipophilic properties of a library of ipsapirone derivatives that were previously synthesized to affect dopamine and serotonin receptors. Lipophilicity indices were determined using computational and chromatographic approaches. In addition, the affinity to human serum albumin (HSA) and phospholipids was assessed using biomimetic chromatography protocols. Quantitative Structure–Retention Relationship (QSRR) methodologies were used to determine the impact of theoretical descriptors on experimentally determined properties. A multiple linear regression (MLR) model was calculated to identify the most important features, and genetic algorithms (GAs) were used to assist in the selection of features. The resultant models showed commendable predictive accuracy, minimal error, and good concordance correlation coefficient values of 0.876, 0.149, and 0.930 for the validation group, respectively.

## 1. Introduction

Numerous drug candidates are dismissed in clinical trials due to insufficient pharmacokinetic properties [1,2]. Therefore, optimizing the physicochemical properties of potential drug molecules at the initial stage of drug development becomes crucial. The optimization is necessary to attain the desired drug metabolism and pharmacokinetic profile in vivo. The lipophilicity of a molecule is a well-known factor that affects its toxicity, absorption, distribution, metabolism, and elimination [3]. Consequently, lipophilicity assessment is one of the basic tests of drug candidates in early drug discovery. The chromatographic approach is the most frequently used among available methods since it offers several advantages compared to the traditional shake-flask procedure. The chromatographic approach requires minimal amounts of sample while being insensitive to impurities, and it is fully automated. In addition, the results of the chromatographic analyses are both repeatable and robust. Therefore, the solid–liquid partitioning methods are highly convenient in the early stages of the drug discovery pipeline, prioritizing high throughput over accuracy [1,4].

The chromatographic approach also allows for measuring other bio-physicochemical properties of given molecules, such as affinity to phospholipids and plasma proteins (PPs) [5]. Plasma protein binding (PPB) mostly affects drug distribution, half-life, and clearance. A molecule bound to PP cannot enter organ tissue via passive diffusion through the physiological barriers. Only unbound drug molecules may interact with therapeutic targets, which means that molecules with high affinity to PPs (above 95%) will show limited brain penetration and low clearance and may cause drug safety issues due to serious drug–drug interactions. Low affinity to PPs, on the other hand, reduces the duration of drug action.

This work assessed the lipophilicity properties of previously synthesized libraries of ipsapirone derivatives designed to affect dopamine (D_2_R) or serotonin receptors (5-HT_1A_R) to reduce the symptoms of depression or schizophrenia [6,7]. In the case of drug candidates targeting the central nervous system (CNS), lipophilicity is an essential property since it determines passive diffusion through the blood–brain barrier (BBB).

Using a chromatographic method, we experimentally determined lipophilicity indices of target ipsapirone derivatives. In parallel, we also calculated the lipophilicity of studied molecules using several computational software. Additionally, the affinity to phospholipids was determined using immobilized artificial membrane (IAM) chromatography. Quantitative Structure–Retention Relationship (QSRR) models were proposed to understand better which molecular descriptors influence their lipophilicity. Furthermore, the affinity to human serum albumin (HSA), which is dominantly plasma protein (PP), was determined, and the relationship between the affinity to HSA and experimental and computational lipophilicity was examined.

## 2. Results and Discussion

### 2.1. Lipophilicity Assessment

Computational approaches for lipophilicity estimation offer several advantages over experimental methods, including quick calculation times and a reduced use of chemical reagents. Moreover, computational methods allow for the prediction of lipophilicity before synthesis, making it relevant to designing potential drug candidates [8]. However, it is important to note that several studies demonstrated that the calculated LogP can differ significantly from the actual value [9,10,11].

In Table 1, calculated lipophilicity indices of functionalized ipsapirone derivatives are summarized. Discrepancies in the computed LogP values are evident across various molecules, with notable differences observed in specific instances: molecule 9 exhibits a substantial variance of 1.87 between the iLogP and Silicos-IT LogP descriptors; molecule 2 shows a difference of 1.75 between iLogP and Silicos-IT LogP; and molecule 19 displays a disparity of 1.72 between MLogP and WlogP.

These variations can be elucidated using the diverse algorithms utilized in the computational methods. In Table 2, the basic descriptors of each algorithm are summarized. The lowest LogP values are derived from the MLogP, reaching the minimum value in 15 instances, while the remaining 11 compounds achieved their lowest values using the Silicos-IT LogP descriptor. The MLogP, or Moriguchi octanol-water partition coefficient, calculated using AlvaDesc software (version 2.0.10), is based on a qualitative structure–logP relationship utilizing topological indices along with molecular properties. The Silicos-IT LogP, calculated using SwissADME (http://www.swissadme.ch, accessed on 1 February 2024), is a hybrid method combining a fragmental approach with a topological one.

Among the considered chemical compounds, molecule 9 was identified as the most hydrophilic substance according to each algorithm. The most lipophilic compound according to six descriptors was molecule 18 the LogP value was the highest for the following specified descriptors: ALogP, LogP99, LogPcons_(AlvaDesc)_, LogP_Chemicalize_, LogP, iLogP, and XLogP3. According to three other descriptors, molecule 19 emerged as the most lipophilic, based on MLogP, WLogP, and LogPcons_(SwissADME)_, and for one descriptor, Silicos-IT LogP, compound **25** proved to be the most lipophilic.

Considering the significant differences in the calculations obtained, the next step of our investigation considered the determination of lipophilicity using a chromatographic approach. Among the available protocols, the fast gradient approach developed by Valko was chosen because it enables the assessment of lipophilicity from a single chromatographic measurement [2,4,12,13,14]. In addition, this approach enables the determination of the acid/basic properties of molecules through the addition of experiments under different pH conditions. A summary of all the chromatographic data is presented in Table 3.

The results show that the investigated ipsapirone derivatives have a rather high lipophilicity when considering the CHI scale from 0 to 100 (extrapolation is allowed). In considering that lipophilicity is a known factor influencing passive diffusion across the BBB, this is an important observation. The affinity to phospholipids can also be determined using one-gradient protocols and the IAM column. CHI_IAM_ can be used as a cut-off point to indicate the potential for promiscuous binding and interference with phospholipids. Among the target structures, only molecule 18 had a slightly higher CHI_IAM_ value of 50.19.

**Figure 1:**
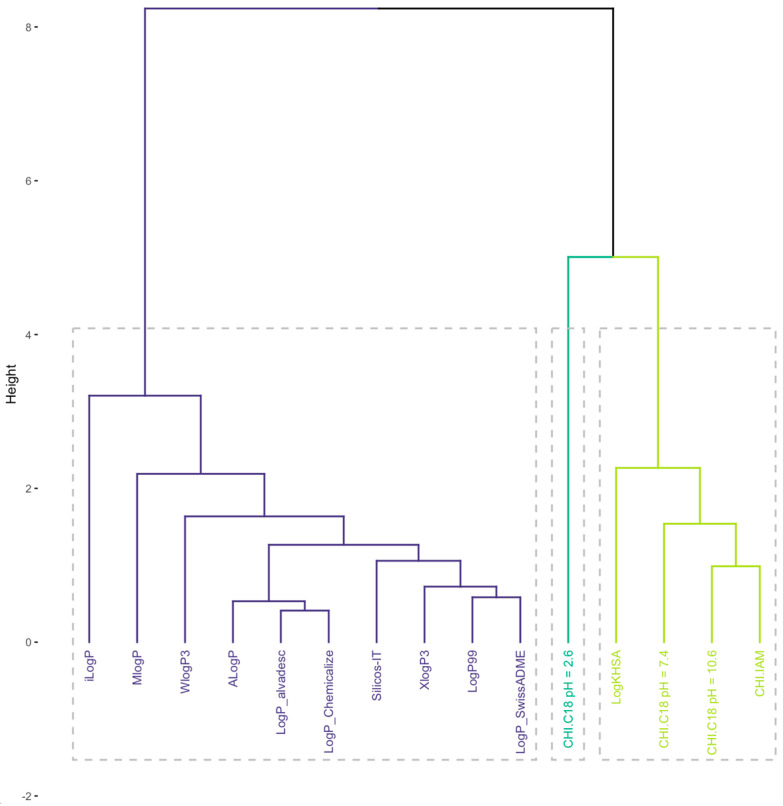
Results of CA analysis.

**Figure 2:**
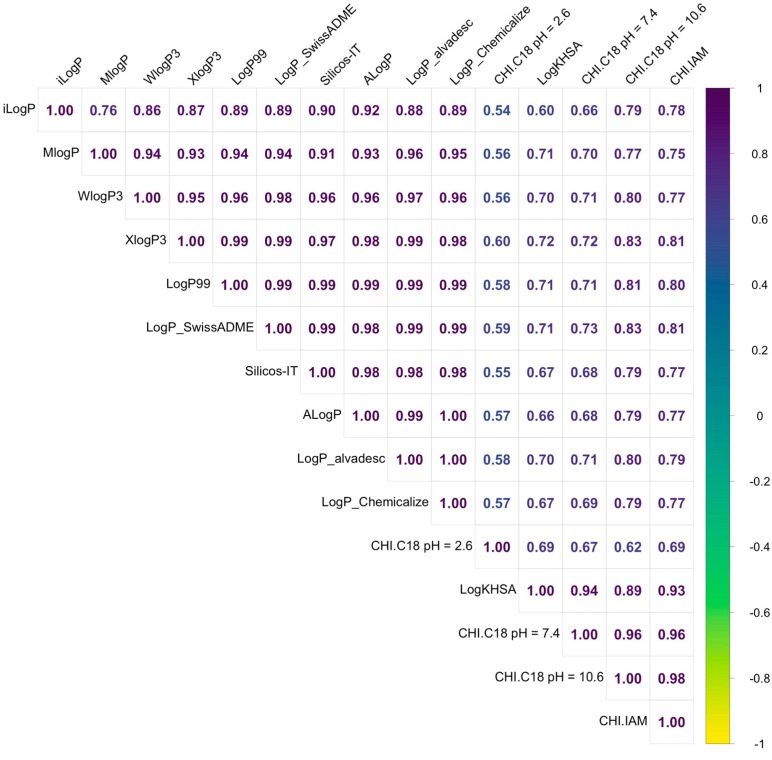
Correlation matrix between calculated and experimentally determined lipophilicity indices and affinity to HSA.

The significantly lower value of CHI under acidic conditions indicates that all ipsapirone derivatives have basic character. Therefore, the CHI under pH_10.6_ can be considered and converted to chromatographically determined LogP, called CHI LogP. A cluster analysis (CA) was performed to compare chromatographically determined and calculated lipophilicity. A CA can be used for the visualization of similarities and differences between studied objects, in this case, lipophilic parameters. The obtained results clearly indicate that significant differences between theoretical and experimental data occurred, and they formed two separate groups (Figure 1). The differences are also visible on the calculated correlation matrix (Figure 2). Moreover, even the more complex algorithms or calculations based on 3D optimized structures do not have significant improvement, and all theoretical descriptors are similarly correlated with CHI LogP (r between 0.77 to 0.83).

### 2.2. QSRR Modeling of Chromatography Determined Lipophilicity and Phospholipophilicity

The following step of our study focuses on QSRR modeling. The QSRR approach, introduced by Kaliszan [15], is currently one of the most widely used and powerful computational methods in the analytical field of chemistry. Numerous QSRR studies have been reported, mainly focused on retention prediction [16], supporting the identification of molecules, mostly in untargeted metabolomics [17], or on the comparison of chromatographic columns and systems [18].

Another advantage of the QSRR approach is the possibility of obtaining insights into the molecular mechanism of retention in the utilized chromatographic system, which can be directly transformed for the relationship between molecule structure and a physicochemical endpoint measured chromatographically [19,20].

Our work focused on the application of QSRR to gain insight into the descriptors that determine the lipophilicity of ipsapirone derivatives. The goal was achieved using a hybrid approach of a genetic algorithm (GA) and multiple linear regression (MLR). In summary, a GA is a stochastic method that assists in solving variable selection problems. Therefore, integrating a GA and MLR may benefit the development of a highly accurate and predictive QSRR model. Table 4 presents a summary of the derived QSRR models together with statistical figures.

Table 5 lists the whole name of each employed molecular descriptor, with its description and assigned block.

The obtained QSRR models indicated some similarities between chromatographically measured lipophilicity and phospholipophilicity. First, in both cases, the LLS_01 descriptor, referring to the local lipophilicity of molecules based on a score derived from the rules proposed by Congreve et al., plays an important role [21]. Furthermore, both models contain CATS descriptors. The CATS descriptors were created by Schneider and are members of the correlation-vector descriptor class, related to the atom-pair descriptor class [22]. The CATS descriptors code the frequencies of atom-type pairings, which may represent possible pharmacophoric locations and are adjusted using the topological characteristics of molecules. The CATS descriptors cover five potential pharmacophore points (PPPs): lipophilic (L), positively charged (P), negatively charged (N), hydrogen-bond acceptor (A), and hydrogen-bond donor (D). Although, in the case of the IAM model, the percentage of N atoms completed the model, and it should be highlighted that all molecules have the same chemical characteristics and are typical organic bases.

**Figure 3:**
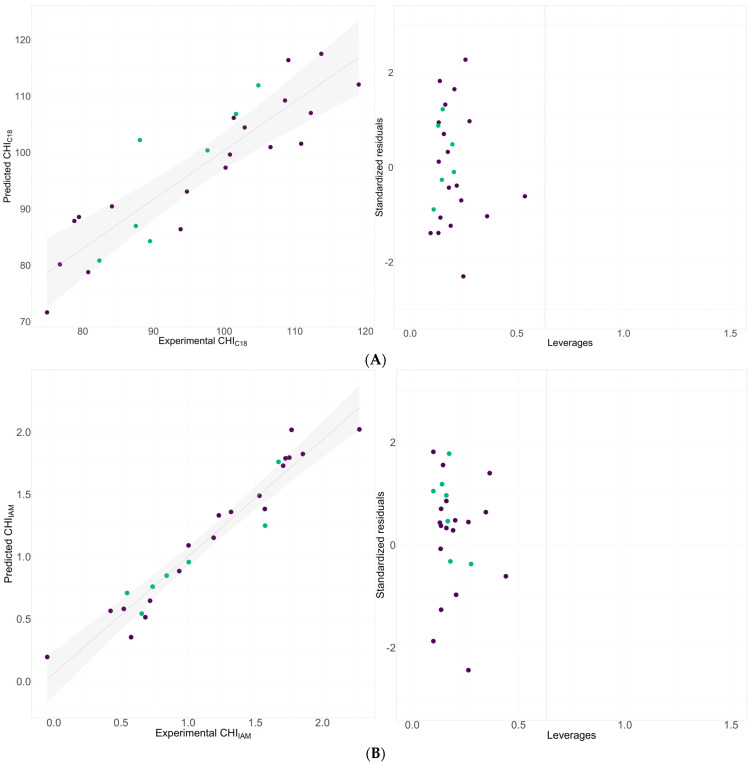
Comparison between experimental retention indices and those predicted by models and Williams plot for (**A**) C_18_-bonded stationary phase using pH 7.4 buffer and (**B**) IAM stationary phase. Green dots refer to the training set, whereas purple ones refer to the validation set.

The models obtained are well fitted, as indicated by the statistical figures of the training and testing sets, including the R^2^, Q^2L^_OO_, and RMSE_TR_, and exhibit appropriate predictive parameters, such as the RMSE_P_. Furthermore, as confirmed through a Williams plot, the proposed model’s applicability domain (AD) indicated good predictions (Figure 3).

### 2.3. Interaction between Plasma Protein

Utilizing columns modified by plasma proteins, such as human serum albumin (HSA), allows for estimating binding to plasma protein (PPB). Generally, PPB affects drug pharmacokinetics, including distribution, half-life, and clearance [23,24,25]. While lipophilicity is a well-known factor in determining PPB, it should also be considered in early drug discovery. In general, more lipophilic compounds tend to have higher percentages of PP due to nonspecific interactions with proteins. However, hydrophilic compounds can also bind strongly to PP through spherical and electrostatic interactions by binding in protein pockets.

Chromatographically determined HS*A* affinity can be expressed as log*K*_HSA_, ranging from −0.8 to 1.9, or recal*c*ulated to a more informative % of HAS binding. Analyzing the structures showed moderate affinity to HAS, except for molecule 23, which is relatively low-binding to HAS (log*K*_HSA_ − 0.05 and %HSA = 47.66), and molecule 18, which showed a higher affinity to reference diclofenac.

**Figure 4:**
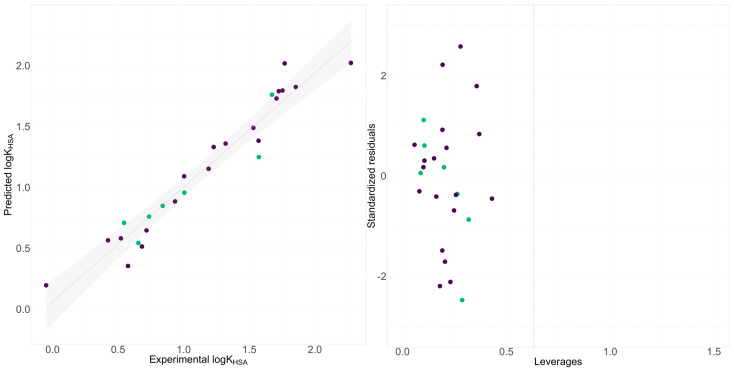
Comparison between the experimental retention indices and those predicted by models and Williams plot for HSA stationary phase for the best-obtained model (model 3). Green dots refer to the training set, whereas purple ones refer to the validation set.

The next step of our investigation focused on finding molecular properties of ipsapirone derivatives that influence the affinity to HSA. Based on computational descriptors, the model yielded predictive statistics above 0.7 (Table S1 in the Supplementary Materials). This can be considered an acceptable model; however, an attempt was made to achieve a model with a more satisfactory result. To model this endpoint, chromatographically determined lipophilicity and phospholipophilicity were used. The CHI_IAM_ descriptor was selected as a significant descriptor based on a GA. The statistics of this model significantly improved, providing more satisfactory results (Figure 4).

The results suggest that the experimental phospholipophilicity data align more closely with the predicted interactions with PP. The correlation matrix, PCA, and HCA (Figure 1) were used to check this suggestion, which indicate that experimental data were more effective for estimating certain biological properties, such as interactions with PP. All investigated methods indicated that experimentally measured lipophilicity or phospholipophilicity are better for predicting interactions with PP since these experiments were grouped together in the CA results.

## 3. Materials and Methods

### 3.1. Solvents

Buffer solutions (polar solvents for HPLC experiments) were prepared by dissolving HPLC-grade ammonium acetate (VWR International, Leuven, Belgium) in ultrapure water obtained from a Milli-Q water purification system (Merck Millipore, Darmstadt, Germany). To adjust the buffer solutions’ pH, two concentrated solutions were used: ammonia (Avantor Performance Materials Poland S.A., Gliwice, Poland) and acetic acid (Chempur, Piekary Śląskie, Poland). HPLC-grade acetonitrile (Chempur, Piekary Śląskie, Poland) and isopropanol (VWR International, Leuven, Belgium) were used as non-polar solvents for HPLC experiments.

### 3.2. Analytes

The compounds studied were the ipsapirone derivatives described in the articles on their pharmacological activity and synthesis [6,7]. All investigated molecules are are listed in the Supplementary Materials (Table S2).

#### Calibration Sets

The reference substances constituting calibration mixtures for HSA-HPLC, RP-HPLC, and IAM-HPLC experiments were obtained from four commercial sources: Alfa Aesar (Haverhill, MA, USA), Sigma-Aldrich (Steinheim, Germany), Cayman Chemical (Ann Arbor, MI, USA), and Acros Organic (Pittsburg, PA, USA) as reported in Table S3.

### 3.3. Chromatographic Analysis

All experiments were performed using a high-performance liquid chromatography system (Shimadzu Prominence LC-2030C 3D) equipped with a DAD detector and controlled through a LabSolution system (version 5.90, Shimadzu, Tokyo, Japan).

For RP-HPLC experiments, a C_18_ Hypersil GOLD^TM^ (50 mm × 4.6 mm; 5.0 µm with a guard column; Thermo Scientific, Waltham, MA, USA) column was applied. The column temperature was set to 40 °C. Three water solutions were used as mobile phase A: acetic acid at pH 2.6, mM ammonium acetate at pH 7.4, and 50 mM ammonium acetate at pH 10.5. Mobile phase B was acetonitrile (ACN). The linear gradient from 2 to 98% ACN was applied from 0 to 5.25 min and held at 98% ACN for 1.75. The mobile phase flow rate was 1.5 mL/min throughout the experiment.

The immobilized artificial membrane column (IAM.PC.DD2, 10 × 4.6 mm × 10.0 µm with a guard column (Regis Technologies; Morton Grove, IL, USA) was used for IAM-HPLC experiments. The column temperature was set to 30 °C. Similar to RP-HPLC, mobile phase A was a 50 mM ammonium acetate at pH 7.4, and mobile phase B was acetonitrile. The linear gradient from 0 to 85% ACN was applied from 0 to 5.25 min and held at 98% ACN for 0.5 min at a constant flow rate of 1.5 mL/min.

For HSA-HPLC experiments, the column was Chiralpak^®^ HSA (100 × 4 mm; 5 µm with safety guard column; Daicel Chiral Technologies, West Chester, PA, USA). The column temperature was set to 30 °C. The same phase A as in the IAM-HPLC case was used, and the mobile phase B was isopropanol (i-PrOH). The linear gradient from 0 to 20% i-PrOH was applied from 0 to 15 min, held at 20% i-PrOH for 12 min, and then returned to pure ammonium acetate solution. The mobile phase flow rate was 0.9 mL/min throughout the experiment. Three minutes of column recalibration was applied between each run. The retention times were collected at wavelengths between 190 and 300 nm, and the injection volume was 10 μL.

### 3.4. Theoretical Descriptors

Theoretical descriptors were calculated using Chemicalize software (https://chemicalize.com, accessed on 1 February 2024) and alvaDesc software (version 2.0.10, Alvascience, Lecco, Italy) based on geometry optimization using Baker’s EigenFollowing method using MOPAC software (version 3.0). Then, constant, almost constant, and highly correlated (r = 0.95) descriptors were removed. The final number of descriptors was 3170. In addition, several lipophilicity indices were calculated for each compound using the SwissADME web application (http://www.swissadme.ch, accessed on 1 February 2024) based on the SMILES notation.

### 3.5. CA Analysis

A CA was performed on databases that included chromatographic data and in silico-calculated lipophilicity indices. In order to eliminate the impact of various lipophilicity scales, data were standardized before analysis. The CA was conducted using Ward’s agglomeration rule and the Euclidian distance measure using a self-written R script.

### 3.6. QSRR Analysis

The process of choosing descriptors was facilitated through the utilization of a genetic algorithm (GA), while a multiple linear regression (MLR) method was applied for a regression analysis using QSARINS version 2.2.4 software, developed by Gramatica et al. [26,27]. The parameters governing the genetic algorithm were determined by specifying a population size of 10, a mutation rate of 20, and 500 generations per size. Prior to the computation of GA-MLR for each modeled endpoint, the target solutes were allocated into distinct groups, comprising a training group (n = 19) and a validation group (n = 7). An analysis of the LogK_HSA_ endpoint was conducted, incorporating two previously considered endpoints, namely CHI_C18_ and CHI_IAM_, as descriptors.

## 4. Conclusions

In summary, our study employed a combined experimental and computational approach to investigate the lipophilicity of ipsapirone derivatives. We observed significant disparities between calculated LogP values from computational methods. Additionally, the differences between calculated and chromatographically established results were observed through a CA. Relatively high lipophilicity indices suggest that the investigated structure should be useful for crossing the BBB. Our QSRR modeling efforts identified key molecular descriptors influencing lipophilicity, phospholipophilicity, and binding to HSA. The models related to lipophilicity and affinity to phospholipids present CATS 3D and drug-like indices as investigated descriptors. What is important is the integration of experimental data into predictive models for plasma protein binding, which improved the model performance, emphasizing the importance of chromatographic assessments.

## Figures and Tables

**Table 1:** The calculated LogP values of the ipsapirone derivatives concerning the computational model.

No.	MLogP	ALogP	LogP99	Log_AlvaDesc_	Log_Chemicalize_	iLogP	XLogP3	WLogP	Silicos-IT	LogP_SwissADME_	CHI LogP *
1	2.15	2.40	2.04	2.20	2.49	2.92	2.57	1.98	1.59	2.19	3.48
2	1.89	2.38	2.05	2.11	2.33	3.40	2.55	1.99	1.65	2.23	4.44
3	2.86	3.72	3.35	3.31	3.70	3.32	3.83	3.29	2.88	3.18	3.27
4	2.64	3.06	2.70	2.80	3.09	3.27	3.2	2.64	2.23	2.74	3.97
5	2.60	3.43	2.82	2.95	3.45	3.36	3.29	2.76	2.37	2.82	3.70
6	2.32	3.41	2.83	2.86	3.29	3.58	3.26	2.77	2.44	2.81	3.49
7	3.29	4.76	4.13	4.06	4.66	4.03	4.54	4.07	3.66	3.86	4.63
8	3.08	4.10	3.48	3.55	4.06	3.71	3.92	3.42	3.01	3.37	3.98
9	1.86	2.17	1.61	1.88	2.21	3.12	1.90	1.55	1.25	1.78	2.91
10	2.43	2.82	2.22	2.49	2.83	3.18	2.55	2.16	1.81	2.28	3.10
11	3.29	4.34	3.98	3.87	4.44	3.77	4.54	3.92	3.40	3.72	4.55
12	2.81	3.89	3.21	3.30	3.90	3.68	3.65	3.15	2.77	3.16	3.98
13	2.53	3.87	3.22	3.21	3.74	3.86	3.62	3.16	2.84	3.14	3.76
14	2.53	3.87	3.22	3.21	3.74	3.92	3.62	3.16	2.84	3.15	3.92
15	2.53	3.87	3.22	3.21	3.74	3.96	3.62	3.16	2.84	3.16	3.76
16	3.29	4.55	3.87	3.90	4.50	3.94	4.27	3.81	3.41	3.69	4.58
17	3.29	4.55	3.87	3.90	4.50	3.92	4.27	3.81	3.41	3.69	4.49
18	3.50	5.22	4.52	4.41	5.10	4.20	4.90	4.46	4.05	4.17	4.93
19	3.60	4.83	4.23	4.22	4.77	4.01	4.53	5.32	3.86	4.21	4.69
20	3.18	4.09	3.35	3.54	4.04	3.70	3.75	3.71	3.19	3.45	4.19
21	3.49	4.80	4.37	4.22	4.89	4.11	4.90	4.31	3.79	4.06	4.91
22	2.65	3.28	2.61	2.84	3.27	3.47	2.91	2.55	2.20	2.61	3.44
23	2.09	2.63	2.00	2.24	2.65	3.33	2.26	1.94	1.64	2.10	3.37
24	3.00	4.64	3.82	3.82	4.56	4.03	4.38	3.76	3.85	3.70	4.33
25	3.16	4.75	4.43	4.11	4.77	4.09	4.66	4.37	4.42	4.12	4.40
26	2.72	3.84	3.36	3.31	3.92	3.54	3.48	3.00	2.81	3.05	3.93

* CHI LogP was calculated using a linear model based on chromatographically determined CHI on nonionized forms; CHI LogP = 0.054 × CHI − 1.467.

**Table 2:** List of software used with information regarding algorithms.

Name	Description	Software
MLogP	Based on quantitative structure–logP relationships, using topological indexes.	AlvaDesc
ALogP	Ghose–Crippen octanol–water partition coefficient	AlvaDesc
LogP99	Wildmann–Crippen octanol–water partition coefficient—atom-based method	AlvaDesc
LogPcons_AlvaDesc_	Consensus model of LogP from AlvaDesc	AlvaDesc
LogP_Chemicalize_	Atomic correction using the contribution of individual molecular fragments	Chemicalize
iLogP	In-house physics-based method relying on free energies of solvation in n-octanol and water calculated using Generalized Born and solvent-accessible surface area (GB/SA) model—atomic- and knowledge-based method	SwissADME
XLogP3	Atomistic method including corrective factors and knowledge-based library—atomistic- and knowledge-based method	SwissADME
WLogP	Implementation of a purely atomistic method based on 27 fragments and 7 topological descriptors—hybrid fragmental/topological method	SwissADME
Silicos-IT	Hybrid method relying on 27 fragments and 7 topological descriptors—hybrid fragmental/topological method	SwissADME
Consensus LogP_SwissADME_	The arithmetic mean of the values predicted using the five propose methods—average of all predictions calculated using SwissADME	SwissADME

**Table 4:** Obtained QSRR models with statistical figures.

Model	Equation					
1	CHI_C18 pH = 7.4_ = 3.375(±1.761)RDF020i + 3.262(±0.456)CATS3D_12_LL − 141.299(±0.842) LLS_01 + 90.055(±20.430)					
2	CHI_IAM_ = 0.872(±0.269)CATS3D_07_AL − 33.834(±0.565)LLS_01 − 1.615(±0.425) N% + 58.328(±6.162)					
3	Log*K*_HSA_ = 0.114(±0.952) CHI_IAM_ − 0.857(±0.141)GATS2e − 0.050(±0.274) RDF155u − 2.751(±0.994)					
	**R^2^**	**RMSE_tr_**	**Q^2^_LOO_**	**R^2^_EXT_**	**RMSE_P_**	**CCC_Ext_**
1	0.838	5.561	0.748	0.751	6.687	0.770
2	0.844	1.944	0.736	0.852	2.019	0.863
3	0.946	0.138	0.910	0.876	0.149	0.930

**Table 5:** Full name and block for molecular descriptors applied in QSRR analysis.

Model	Name	Description	Block
1	RDF020i	Radial Distribution Function—020/weighted using ionization potential	RDF descriptors
	CATS3D_12_LL	CATS3D Lipophilic-Lipophilic BIN 12 (12.000–13.000 Å)	CATS 3D
	LLS_01	modified lead-like score	Drug-like indices
2	CATS3D_07_AL	CATS3D Acceptor-Lipophilic BIN 07 (7.000–8.000 Å)	CATS 3D
	LLS_01	modified lead-like score	Drug-like indices
	N%	percentage of N atoms	Constitutional indices
3	CHI IAM	CHIIAM values from the experiment	Experiment
	GATS2e	Geary autocorrelation of lag 2 weighted using Sanderson electronegativity	2D autocorrelations
	RDF155u	Radial Distribution Function—155/unweighted	RDF descriptors

**Table 3:** The summarized values of the chromatographic and biochromatographic indices of the target functionalized ipsapirone derivatives.

No.	CHI_C18 pH = 2.6_	CHI_C18 pH = 7.4_	CHI_C18 pH = 10.6_	CHI_IAM_	Log*K*_HSA_	%HSA
1	48.96	88.07	91.64	36.93	0.74	85.32
2	60.08	110.90	109.41	46.90	1.72	99.13
3	49.19	78.80	87.69	35.71	0.52	77.60
4	56.31	100.82	100.70	42.61	1.32	96.38
5	52.20	89.50	95.60	39.09	0.84	88.23
6	52.94	79.45	91.77	38.03	0.68	83.59
7	72.79	112.26	112.92	48.28	1.77	99.31
8	97.99	101.36	100.81	44.30	1.53	98.11
9	48.71	74.93	81.12	34.40	0.58	79.78
10	38.96	76.74	84.51	35.73	0.72	84.71
11	63.51	104.83	111.43	47.26	1.67	98.90
12	55.26	93.84	100.78	41.59	1.00	91.87
13	54.89	84.12	96.78	40.54	0.42	73.28
14	55.41	94.73	99.75	41.42	0.93	90.46
15	55.04	87.50	96.78	40.04	0.65	82.67
16	61.89	108.61	111.92	46.73	1.71	99.06
17	61.34	106.56	110.38	46.32	1.57	98.36
18	65.39	119.04	118.39	50.19	2.27	<99.80
19	63.86	113.75	114.05	46.84	1.85	99.61
20	57.21	97.64	104.68	42.69	1.00	91.90
21	65.07	109.08	118.03	48.85	1.75	99.25
22	43.25	82.33	90.87	38.48	0.55	78.60
23	41.03	80.76	89.54	34.77	−0.05	47.66
24	60.18	102.90	107.33	45.78	1.23	95.36
25	60.65	101.68	108.64	45.96	1.57	98.37
26	55.76	100.20	100.01	41.48	1.19	94.87

## Data Availability

Data will be available on Zenodo.

## Supplementary Materials

The following supporting information can be downloaded at https://www.mdpi.com/article/10.3390/molecules29081862/s1: Table S1. Summary of QSRR model of HSA based on the theoretical descriptors; Table S2. List and SMILE notation of target structures; Table S3. Calibration mixtures for biomimetic chromatography.
