# Supplementary material for: Evaluation of Physicochemical Properties of Ipsapirone Derivatives Based on Chromatographic and Chemometric Approaches
Source: Molecules. 2024 Apr 19;29(8):1862. doi: 10.3390/molecules29081862 (PMC11054528; doi:10.3390/molecules29081862)
Supplement: Supplementary file 1 [file molecules-29-01862-s001.zip › molecules-2965266-supplementary.pdf]

molecules

Supplementary materials

Evaluation of Physicochemical Properties of Ipsapirone Derivatives Based on Chromatographic and Chemometric Approaches

Wiktor Nisterenko [1], Damian Kułaga [2], Mateusz Woziński [1], Yash Raj Singh [3], Beata Judzińska [4,5], Karolina Jagiello [4,5], Katarzyna Ewa Greber [1], Wiesław Sawicki [1] and Krzesimir Ciura [5,*]

[1] Department of Physical Chemistry, Faculty of Pharmacy, Medical University of Gdańsk, Aleja Generała Józefa Hallera 107, 80-416 Gdańsk, Poland; wnisterenko@gmail.com (W.N.); mateusz.wozinski@gumed.edu.pl (M.W.); katarzyna.greber@gumed.edu.pl (K.E.G.); w.sawicki@gumed.edu.pl (W.S.)
[2] Department of Organic Chemistry and Technology, Faculty of Chemical Engineering and Technology, Cracow University of Technology, 24 Warszawska Street, 31-155 Cracow, Poland; damian.kulaga@pk.edu.pl
[3] Department of Pharmaceutical Quality Assurance, LJ Institute of Pharmacy, LJ University, Ahmedabad 382210, India; yashraj.0804@gmail.com
[4] QSAR Lab, Trzy Lipy 3, 80-172 Gdańsk, Poland; b.judzinska@qsarlab.com (B.J.); k.jagiello@qsarlab.com (K.J.)
[5] Laboratory of Environmental Chemoinformatics, Faculty of Chemistry, University of Gdansk, Wita Stwosza 63, 80-308 Gdansk, Poland
* Correspondence: krzesimir.ciura@gumed.edu.pl

Table S1. Summary of QSRR model of HSA based on the theoretical descriptors.

**QSRR equation**						
$logK_{HSA}$ = 0.230(±0.335) F05[C-N] 0.060(±0.330) RDF155u 5.899(±0.821) LLS_01 + 1.955(±1.240)						
**Eq.**	**R^2**	**$RMSE_{tr}$**	**Q^2_{LLO}**	**R^2_{EXT}**	**$RMSE_{Ext}$**	**CCC_{Ext}**
1	0.786	0.274	0.670	0.7093	0.302	0.7954

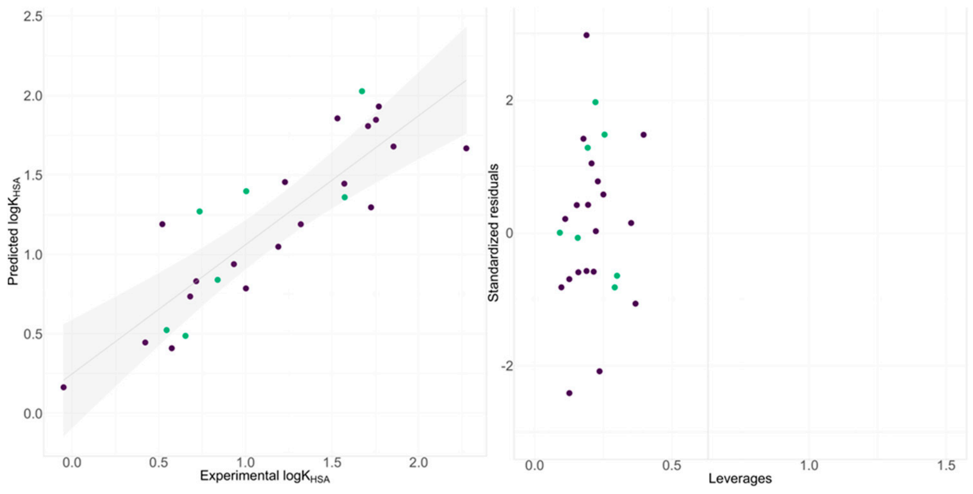

Table S2. List and SMILE notation of target structures.

No	Smiles	linker	R
1	O=C1N(CCCN2CCN(C3=CC=CC=C3)CC2)S(C4=CC=CC=C41)(=O)=O	butyl	H
2	O=C1N(CCCN2CCN(C3=CC=CC=C3OC)CC2)S(C4=CC=CC=C41)(=O)=O	butyl	2-OMe-Ph
3	O=C1N(CCCN2CCN(C3=CC=CC(Cl)=C3Cl)CC2)S(C4=CC=CC=C41)(=O)=O	butyl	2,3-diCl-Ph
4	O=C1N(CCCN2CCN(C3=CC=C(Cl)C=C3)CC2)S(C4=CC=CC=C41)(=O)=O	butyl	4-Cl-Ph
5	O=C1N(CCCCCN2CCN(C3=CC=CC=C3)CC2)S(C4=CC=CC=C41)(=O)=O	pentyl	H
6	O=C1N(CCCCCN2CCN(C3=CC=CC=C3OC)CC2)S(C4=CC=CC=C41)(=O)=O	pentyl	2-OMe-Ph
7	O=C1N(CCCCCN2CCN(C3=CC=CC(Cl)=C3Cl)CC2)S(C4=CC=CC=C41)(=O)=O	pentyl	2,3-diCl-Ph
8	O=C1N(CCCCCN2CCN(C3=CC=C(Cl)C=C3)CC2)S(C4=CC=CC=C41)(=O)=O	pentyl	4-Cl-Ph
9	O=C1N(CCCCCN2CCN(C3=NC=CC=N3)CC2)S(C4=CC=CC=C41)(=O)=O	pentyl	2-pirymidyl
10	O=C1N(CCCCCN2CCN(C3=CC=CC=N3)CC2)S(C4=CC=CC=C41)(=O)=O	pentyl	2-pirydyl
11	O=C1N(CCCCCN2CCN(C3=C(C=CC=C4)C4=CC=C3)CC2)S(C5=CC=CC=C51)(=O)=O	pentyl	1-naphthyl
12	O=C1N(CCCCCCN2CCN(C3=CC=CC=C3)CC2)S(C4=CC=CC=C41)(=O)=O	heksyl	H
13	O=C1N(CCCCCCN2CCN(C3=CC=CC=C3OC)CC2)S(C4=CC=CC=C41)(=O)=O	heksyl	2-OMe-Ph
14	O=C1N(CCCCCCN2CCN(C3=CC(OC)=CC=C3)CC2)S(C4=CC=CC=C41)(=O)=O	heksyl	3-OMe-Ph
15	O=C1N(CCCCCCN2CCN(C3=CC=C(OC)C=C3)CC2)S(C4=CC=CC=C41)(=O)=O	heksyl	4-OMe-Ph
16	O=C1N(CCCCCCN2CCN(C3=CC=CC(Cl)=C3)CC2)S(C4=CC=CC=C41)(=O)=O	heksyl	3-Cl-Ph
17	O=C1N(CCCCCCN2CCN(C3=CC=C(Cl)C=C3)CC2)S(C4=CC=CC=C41)(=O)=O	heksyl	4-Cl-Ph
18	O=C1N(CCCCCCN2CCN(C3=CC=C(Cl)C(Cl)=C3)CC2)S(C4=CC=CC=C41)(=O)=O	heksyl	3,4-diCl-Ph
19	O=C1N(CCCCCCN2CCN(C3=CC=CC(C(F)(F)F)=C3)CC2)S(C4=CC=CC=C41)(=O)=O	heksyl	3-CF_3-Ph
20	O=C1N(CCCCCCN2CCN(C3=CC=CC=C3F)CC2)S(C4=CC=CC=C41)(=O)=O	heksyl	2-F-Ph
21	O=C1N(CCCCCCN2CCN(C3=C(C=CC=C4)C4=CC=C3)CC2)S(C5=CC=CC=C51)(=O)=O	heksyl	1-naphthyl
22	O=C1N(CCCCCCN2CCN(C3=CC=CC=N3)CC2)S(C4=CC=CC=C41)(=O)=O	heksyl	1-pirydyl
23	O=C1N(CCCCCCN2CCN(C3=NC=CC=N3)CC2)S(C4=CC=CC=C41)(=O)=O	heksyl	1-pirymidynyl
24	O=C1N(CCCCCCN2CCN(C3=NSC4=C3C=CC=C4)CC2)S(C5=CC=CC=C51)(=O)=O	heksyl	3-benzisothiazole
25	O=C1N(CCCCCCN2CCN(C3=CC=CC4=C3C=CS4)CC2)S(C5=CC=CC=C51)(=O)=O	heksyl	1-benzothiophen
26	O=C1N(CC2=CC=C(CN3CCN(C4=CC=CC=C4OC)CC3)C=C2)S(C5=CC=CC=C51)(=O)=O	xylene	H

General structure of investigated molecules

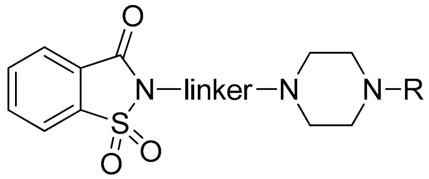

Table S3. Calibration mixtures for biomimetic chromatography.

**Compound**	t_{mean}	**SD**	**CHI** $_{C18}$
	**C_{18}-HPLC**		
Theophylline	2.19	0.024	18.4
Benzimidazole	2.60	0.009	34.3
Colichicine	3.27	0.006	42.0
Acetophenone	3.58	0.007	65.1
Indole	3.99	0.005	71.5
Propiophenone	4.13	0.005	77.5
Butyrophenone	4.54	0.006	87.5
Valerophenone	4.91	0.006	96.2
	**IAM-HPLC**		
Paracetamol	2.27	0.075	2.9
Acetanilidine	2.84	0.047	11.5
Acetophenone	3.16	0.038	17.2
Propiohenone	3.79	0.023	25.9
Butyrophenone	4.26	0.015	32.0
Valerophenone	4.65	0.011	37.3
Hexanophenone	4.98	0.010	41.8
Heptanophenone	5.25	0.010	45.7
Octanophenone	5.49	0.010	49.4
	**HSA-HPLC**		
Nizatidine	2.10	0.027	35.0%
Carbamazepine	5.31	0.069	75.0%
Nicardipine	11.09	0.216	95.0%
Indometacine	20.80	0.010	99.0%
Diclofenac	33.67	0.256	99.8%
